# Cuban Brown Propolis Interferes in the Crosstalk between Colorectal Cancer Cells and M2 Macrophages

**DOI:** 10.3390/nu12072040

**Published:** 2020-07-09

**Authors:** Yahima Frión-Herrera, Daniela Gabbia, Michela Scaffidi, Letizia Zagni, Osmany Cuesta-Rubio, Sara De Martin, Maria Carrara

**Affiliations:** 1 Department of Pharmaceutical and Pharmacological Sciences, University of Padova, L.go Meneghetti 2, 35131 Padova, Italy; yahima81@gmail.com (Y.F.-H.); daniela.gabbia@unipd.it (D.G.); michela.scaffidi@studenti.unipd.it (M.S.); letizia.zagni@studenti.unipd.it (L.Z.); maria.carrara@unipd.it (M.C.); 2 Chemistry and Health Faculty, Technical University of Machala, Ave. Panamericana Vía a Pasaje Km. 5 1/2, Machala 070101, Ecuador; osmanycuesta@yahoo.com

**Keywords:** propolis, nemorosone, colorectal cancer, tumor-associated macrophages, epithelial–mesenchymal transition

## Abstract

Tumor-associated macrophages (TAMs), primarily the M2 phenotype, are involved in the progression and metastasis of colorectal cancer (CRC). Cuban brown propolis (Cp) and its main component Nemorosone (Nem) displays an antiproliferative effect on different cancer cells, including CRC cell lines. However, whether Cp and Nem could exploit its effect on CRC cells by targeting their relationship with TAMs remains to be elucidated. In this study, we differentiated the human monocytic THP-1 cells to M2 macrophages and confirmed this transition by immunofluorescence (IF) staining, qRT-PCR and zymography. An MTT assay was performed to determine the effect of Cp and Nem on the viability of CRC HT-29 cells co-cultured with M2 macrophages. Furthermore, the migration and invasion abilities of HT-29 cells were determined by Transwell assays and the expression levels of epithelial–mesenchymal transition (EMT) markers were analyzed by IF staining. We demonstrated that Cp and Nem reduced the viability of M2 macrophages and, accordingly, the activity of the MMP-9 metalloprotein. Moreover, we demonstrated that M2 macrophages produce soluble factors that positively regulate HT-29 cell growth, migration and invasion. These M2-mediated effects were counteracted by Cp and Nem treatments, which also played a role in regulating the expression of the EMT markers E-cadherin and vimentin. Taken together, our results indicate that Nem contained in Cp interferes in the crosstalk between CRC cells and TAMs, by targeting M2 macrophages.

## 1. Introduction

Tumor-associated macrophages (TAMs) play an important role in the creation of a microenvironment favorable for tumor growth and the development of metastases in patients with colorectal cancer (CRC) [1,2]. Owing to the key role of TAMs in the orchestration of the tumor microenvironment (TME), they are an important therapeutic target for cancer treatment [3].

TAMs include both M1- and M2-macrophages, according to their functional characteristics. Since M1 macrophages produce immunostimulatory and proinflammatory cytokines, they are characterized by a strong antitumor activity [4]. By contrast, the M2 phenotype produces anti-inflammatory cytokines, promotes tumor progression and inhibits the immunological recognition of CRC cells, thereby stimulating cell growth, invasion and metastasis [1]. Therefore, targeting and suppressing M2 activity and inhibiting the crosstalk between CRC cells and TAMs are therapeutic anticancer strategies that could be exploited.

Several studies have demonstrated that natural products can influence the polarization and activity of M2 macrophages, besides displaying antitumor properties [5]. In this context, the number of studies evaluating the effect of natural compounds on the relationship between TAMs and tumor cells in TME is currently increasing [6].

Bee products have been extensively evaluated for their effect on the proliferation of different cancer cells [7,8,9]. Furthermore, it has been demonstrated that propolis, a natural product produced by honeybee, has an interesting anticancer potential according to its chemical composition [10,11], which essentially depends on the geographical zone of collection [12]. Caribbean propolis from Cuba are identified as brown, yellow and red propolis [13], the first one being the most studied due to its content of Nemorosone (Nem). This prenylated benzophenone is considered the main active component of Cuban brown propolis (Cp) [14,15,16]. Recent experimental studies have shown that Cp and Nem display an antiproliferative and cytotoxic activity on different CRC cells [15,17]. However, the effect of Cp and Nem on the crosstalk between macrophages and these cells remains to be evaluated.

In the light of these considerations, the aim of the present study is investigating the effect of Nem and two different samples of Cuban brown propolis (named Cp5 and Cp17), which have already been chemically characterized [13,15], on the relationship between M2-like macrophages and the CRC cell line HT-29. The results obtained from this study provided new evidences regarding the biologic activity of Cp and Nem, suggesting the evaluation of their employment as novel agents for CRC treatment via modulation of the TME.

## 2. Materials and Methods

### 2.1. Reagents and Antibodies

Roswell Park Memorial Institute (RPMI) 1640 medium was purchased from Mediatech (Manassas, VA, USA), Dulbecco’s modified eagle medium (DMEM) and Fetal bovine serum (FBS) were purchased from BioWhittaker Reagents Lonza (Belgium, UK). Dulbecco’s phosphate-buffered saline (DPBS), trypsin-EDTA, l-glutamine were purchased from EuroClone (Milano, Italy). penicillin/streptomycin, dimethyl sulfoxide (DMSO), 3-(4, 5-dimethylthiazol-2-yl)-2, 5-diphenyltetrazolium bromide (MTT), gelatin, crystal violet, lipopolysaccharide (LPS), paraformaldehyde (PFA), propidium iodide and ethanol were purchased from Sigma Aldrich (San Luis, CO, USA). Matrigel matrix was supplied by Corning (Lowell, MA, USA). Cuban brown propolis (Cp5 and Cp17), which had been chemically characterized by spectroscopic techniques [11], were obtained from Estación Experimental Apícola (Havana, Cuba). Nemorosone (1-benzoyl-4-hidroxy-8,8-dimethyl-3,5,7-tris (3-methyl-2-butenyl)-bicyclo-[3.3.1]-non-3-ene-2,9-dione) was provided by the Pharmacy and Food Institute (Havana, Cuba). phorbol-12-myristate-13-acetate (PMA), interleukin 4 (IL-4, hBA-129: sc-4595) and interleukin 13 (IL-13, hBA-114: sc-4606) were purchased from Santa Cruz Biotechnology (Santa Cruz, CA, USA). Hoechst was purchased from Abcam (Cambridge, UK).

Antibodies: Anti-CD86 (bs-1035R-TR) and anti-E-cadherin (bs-10009R-TR) were provided by Bioss Antibodies (Boston, MA, USA). Anti-CD68 (ab31630) was purchased from Abcam (Cambridge, UK). Anti-CD163 (sc-33715), anti CD206 (sc-376108) and anti-vimentin (sc-6260) were obtained from Santa Cruz Biotechnology (Santa Cruz, CA, USA). The secondary antibodies anti-rabbit Alexa-Fluor 488-conjugated and anti-mouse Alexa-Fluor 568-conjugated were purchased from Abcam (Cambridge, UK).

### 2.2. Cell Culture and Sample Preparation

The experiments described in this study were performed using two different human cell lines, i.e., the monocytic THP-1 (ATCC^®^ TIB-202™) and the colorectal adenocarcinoma HT-29 (ATCC^®^ HTB-38™) cells. THP-1 and HT-29 were maintained in RPMI and DMEM media, respectively, with 10% FBS, 1% l-glutamine and 1% penicillin/streptomycin. The stock solutions of Nem and Cp extracts (Cp5 and Cp17) were prepared according to previously published methods [15]. The concentration of Nem in each extract (Cp5, 21.7 μg/mg extract and Cp17, 33.8 μg/mg extract) had been previously determined [15,18] and has been confirmed by our research group.

### 2.3. Macrophage Differentiation

M1- and M2-like macrophages were obtained from THP-1 cells, by incubating them first with 320-nM PMA for 24 h, to obtain differentiated MΦ macrophages, and then for another 48 h with LPS (20 ng/mL) to obtain the M1 phenotype [19], or IL-4 and IL-13 (20 ng/mL) to obtain the M2 phenotype [20]. The expression of the membrane markers CD68, CD86, CD163 and CD206 in THP-1 cells, MΦ, M1 and M2 macrophages was analyzed by immunofluorescence (IF) coupled to confocal microscopy, and the mRNA levels of *IL6*, *IL8*, *TNFα*, *CCL5*, *IL10*, *CCL2* and *VEGF* were measured by quantitative PCR (qRT-PCR).

### 2.4. M2-Conditioned Medium

After THP-1 differentiation, M2-like macrophages were washed three times with fresh medium and cultured for 24 h with RPMI without FBS supplementation. The supernatant of M2 cells (conditioned medium, M2-CM) was collected, centrifuged, filtrated to remove cellular debris, and stored at 20 °C until its use in further experiments.

### 2.5. Immunofluorescence Staining

THP-1 cells, MΦ, M1 or M2-macrophages were grown on glass coverslips in 24-well plates (10 × 10^4^ cells/mL). Before the staining, they were fixed with 4% paraformaldehyde for 30 min [21] and washed with PBS. Unspecific binding sites were blocked by the incubation with 10% FBS for 20 min. The cells were then incubated overnight with either anti-CD68, anti-CD86, anti-CD163 or anti-CD206 antibody (all diluted 1:200) at 4 °C. The fluorescent signals were detected using either anti-mouse Alexa Fluor 568-conjugated or anti-rabbit Alexa Fluor 488-conjugated (both diluted 1:200) secondary antibodies. Nuclei were stained with the blue dye Hoechst 33342. The images were obtained by means of a Zeiss LSM 800 confocal microscope (Zeiss, Milan, Italy) and the Image J software (ver. 1.52t) was used to quantify the intensity of the fluorescent signal [22].

### 2.6. Quantitative Real-Time PCR

THP-1 cells, MΦ, M1 and M2 macrophages were grown in 12-well plates (10 × 10^5^ cells/mL). Total RNA was extracted using the Isolate II RNA kit (Bioline, London, UK), following the manufacturers’ instructions. mRNA levels were measured by means of the One Step SYBR PrimeScript RT-PCR Kit (Takara, Mountain View, CA, USA). *Glyceraldehyde-3-phosphatedehydrogenase (GAPDH)* was used as housekeeping gene, and the mRNA relative expression of the genes of interest was calculated by the 2^−ΔΔCt^ method [23]. The primers used in this study are listed in Table S1.

### 2.7. Cell Treatment

M2-like macrophages and HT-29 cells were treated with Nem (5, 10, 25 and 50 μM), Cp5 (6.25, 12.5, 25, 50, 100 μg/mL) and Cp17 (6.25, 12.5, 25, 50, 100 μg/mL). Additionally, HT-29 cells treated with M2-CM and co-cultures of THP-1 and HT-29 were incubated with the same increasing concentrations of Nem, Cp5 and Cp17 (Figure S1). The concentration of Nem in each propolis sample is indicated in Table 1 [15].

### 2.8. Cell Viability Assay

Cell viability of M2-like macrophages and HT-29 cells was evaluated by means of the MTT assay. Briefly, the cells (10 × 10^4^ cells/mL) were seeded into 96-well plates overnight and incubated with the concentrations of Nem, Cp5 and Cp17 indicated above. Cell viability was determined as described previously [24]. The 50% inhibitory concentrations (IC_50_) were calculated by the appropriate nonlinear regression fit, by means of the GraphPad Prism software ver. 8.0.

### 2.9. Gelatin Zymography

The FBS free-supernatants of M2-like macrophages previously treated with Nem, Cp5 or Cp17 for 24 h, were run for 90 min into a polyacrylamide gel (8%) containing gelatin (1%). At the end of the electrophoresis, the gels were washed twice in 2.5% Triton X-100, incubated with the developing buffer [25], stained with Coomassie blue R-250 and de-stained with a solution containing acetic acid (5%) and methanol (10%). The quantification of the bands was performed by means of the ImageJ software.

### 2.10. Cell Cycle Arrest

After THP-1 cell differentiation, M2-like macrophages (10 × 10^5^ cell/mL, 12-well plates) were treated for 24 h with Nem, Cp5 or Cp17. The cells were fixed in ethanol (70%) for 15 min at 4 °C followed by centrifugation (1800 rpm) for 5 min and stained with propidium iodide (PI, 300 μL) for 30 min. DNA content and cell cycle distribution were determined using Epics XL flow cytometer and CXP software (Beckmann, San Diego, CA, USA).

### 2.11. M2-Like Macrophage and HT-29 Co-Culture

To set up the co-culture system, the M2-like macrophages (10 × 10^4^ cell/mL) were seeded in the upper insert with a 0.4 μm pore size (Corning, Inc., Lowell, MA, USA) and co-cultured with HT-29 cells (10 × 10^4^ cells/mL) in 24-well plates for 24 h, avoiding the direct contact between the two cell lines. At the end of the incubation, the upper inserts were discarded, whereas HT-29 cells were washed and used for further experiments.

### 2.12. Transwell Migration and Invasion Assays

Migration and invasion abilities of HT-29 cells were evaluated by the two-chamber assay (Transwell co-culture system, 24 wells/8 μm pore size, Corning Inc., Lowell, MA, USA) [17]. Transwell upper inserts were coated with Matrigel according to the manufacturer’s instructions. HT-29 cells (10 × 10^4^ cells/mL—upper insert) were co-culture with M2-like macrophages (10 × 10^4^ cell/mL—bottom) for 24 h, and then incubated either in serum-free medium (DMEM) or complete medium (DMEM+10% FBS) with or without Nem, Cp5 or Cp17 in the lower chamber. After 24 h of incubation, the cells still present on the upper surface were removed with a cotton swab. The migrated or invading cells were fixed with PFA (4%) and stained with crystal violet (0.2%). The quantification of cells which underwent migration or invasion was performed by a Nikon T-s microscope(Nikon, Tokyo, Japan) (10× magnification) and the Image J software.

### 2.13. Analysis of Epithelial-Mesenchymal Transition (EMT) Markers

After 24 h of co-culture with M2-like macrophages (10 × 10^4^ cells/mL—upper insert, 0.4 μm pore size Corning, Inc., Lowell, MA, USA), HT-29 cells (10 × 10^4^ cells/mL—bottom) were treated with Nem, Cp5 or Cp17 for 24 h. The expression of EMT markers E-cadherin and vimentin was analyzed by IF staining as described above. Anti-E-cadherin and anti-vimentin (dilution 1:200) were used as primary antibodies, whereas anti-rabbit Alexa Fluor 488 and anti-mouse Alexa Fluor 568 (dilution 1:200) were used as secondary antibodies. Cell nuclei were stained with the blue dye Hoechst 33,342.

### 2.14. Statistical Analysis

All statistical analyses were performed by means of the GraphPad Prism software ver. 8.0 (San Diego, CA, USA). One-way analysis of variance (ANOVA) followed by the post hoc Tukey’s multiple comparisons test was used to assess the eventual significance of the differences observed between treatments. All the experiments were performed in triplicate. *p* < 0.05 was considered statistically significant. If not otherwise stated, data are presented as mean ± SD.

## 3. Results

### 3.1. Differentiation of THP-1 Cells into M2-Like Macrophages

**Figure 1:**
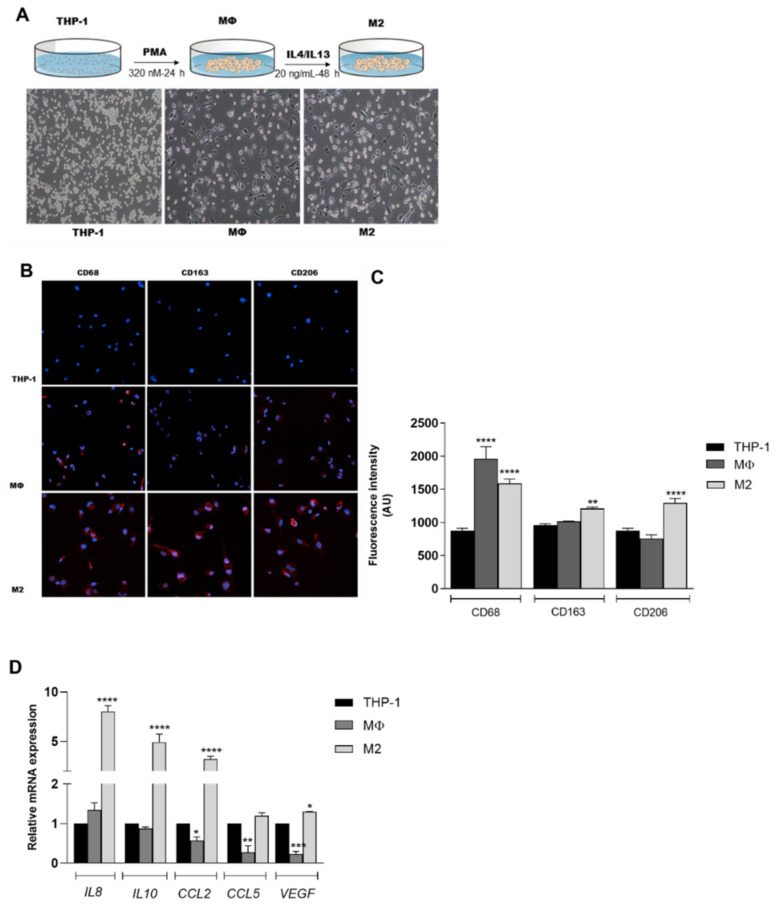
Differentiation of THP-1 cells into macrophages. (**A**) Cell morphology and protocols used to obtain the MΦ and M2 macrophages. Magnification: 10×; (**B**,**C**) immunofluorescence images. CD68, CD163 and CD206 are stained in red. Nuclei are stained blue with Hoechst. Magnification: 20×. A.U. arbitrary units; (**D**) mRNA levels of *IL8, IL10, CCL2, CCL5 and VEGF*. Data are reported as mean ± SD of three independent experiments performed in triplicate. * *p* < 0.05, ** *p* < 0.01, *** *p* < 0.001, **** *p* < 0.0001 vs. THP-1 cells.

As shown in Figure 1A, THP-1 cells showed an adherent and amoeboid morphology after incubation with PMA, IL-4 and IL-13. Accordingly, the increased expression of the surface markers CD68, CD163 and CD206 (Figure 1B,C) confirmed their differentiation into M2 macrophages. Furthermore, we found that selected genes associated with the M2-like phenotype, including *IL8*, *IL10*, *CCL2* and *VEGF* [26,27] were significantly upregulated in these cells with respect to THP-1 cells and MΦ macrophages (Figure 1D). No difference was observed in *CCL5* expression between M2-like macrophages and control cells. Taken together, these data indicate that THP-1 cells were successfully differentiated in vitro into M2-like macrophages.

### 3.2. Effect of Nemorosone and Cuban Propolis on M2-Like Macrophages

**Figure 2:**
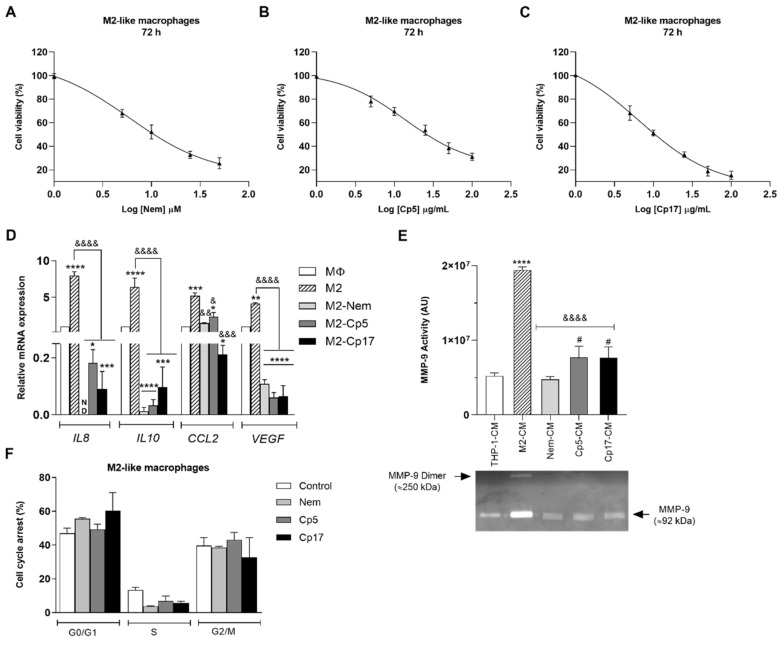
Effect of Nem, Cp5 and Cp17 on M2-like macrophages. (**A**–**C**) MTT assay: M2-like macrophages were exposed to increasing concentrations of Nem (**A**), Cp5 (**B**) and Cp17 (**C**) for 24, 48 and 72 h; (**D**) mRNA levels of IL8, IL10, CCL2 and VEGF after 24 h of exposure to 21 µM, 44 µg/mL, 16 µg/mL of Nem, Cp5 and, Cp17, respectively (IC_50_/72 h); (**E**) MMP-9 activity was evaluated by zymography in different supernatants (THP-1-CM, M2-CM, Nem-CM, Cp5-CM and Cp17-CM); (**F**) Cell cycle analysis after 24 h of exposure to 21 µM, 44 µg/mL, 16 µg/mL of Nem, Cp5 and, Cp17, respectively (IC_50_/72 h). Data are reported as the mean ± SD of three independent experiments. * *p* < 0.05, ** *p* < 0.001, *** *p* < 0.0001, **** *p* < 0.0001 vs. MΦ or THP-1-CM, ^&^ *p* < 0.05, ^&&^ *p* < 0.01, ^&&&^ p < 0.001, ^&&&&^ *p* < 0.0001 vs. M2-like macrophages or M2-CM, ^#^ *p* < 0.05 vs. Nem-CM. N.D. Not detected. A.U. arbitrary units.

**Figure 3:**
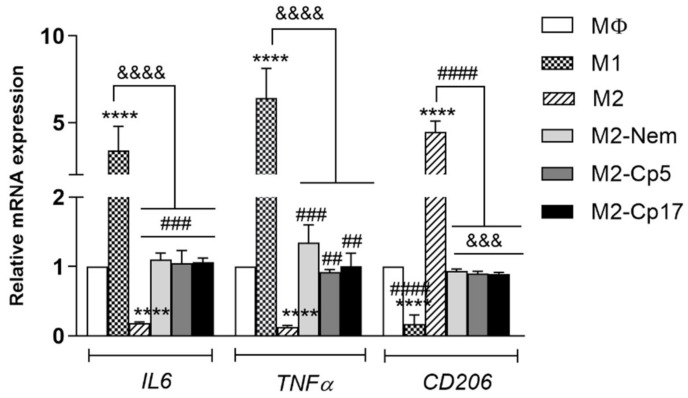
Effect of Nem and Cp on M2 repolarization. mRNA levels of *IL6, TNFα and CD206* after exposure to 21 µM, 44 µg/mL, 16 µg/mL of Nem, Cp5 and, Cp17, respectively. Data reported as the mean ± SD of three independent experiments. **** *p* < 0.0001 vs. MΦ, ^&&&^ *p*< 0.001, ^&&&&^ *p* < 0.0001 vs. M1-like macrophages and ^##^ *p*< 0.01, ^###^ *p* < 0.001, ^####^ *p* < 0.0001 vs. M2-like macrophages.

Since the infiltration of macrophages, in particular those belonging to the M2-phenotype, has been correlated with poor prognosis in different types of cancer, we first evaluated the effect of Nem, Cp5 and Cp17 on the viability of M2 macrophages by means of the MTT assay. As shown in Figure 2A–C and in Table 2, all these treatments reduced cell growth in a dose-dependent manner. Based on these findings, to ensure a cell survival of over 50%, in the experiments described below (Figure 2 and Figure 3) we treated the cells for 24 h with a concentration of Nem, Cp5 and Cp17 equal to the IC_50_ value after 72 h of incubation (Table 2). In these experimental conditions, we observed that the mRNA levels of the cytokines *IL8*, *IL10*, *CCL2* and *VEGF* significantly dropped when M2-like macrophages were treated with Nem, Cp5 and Cp17 (Figure 2D). To further confirm the effect of the treatments on M2 macrophages, we evaluated the activity of MMP-9. This metalloprotein, which is released by M2-like macrophages, is known to contribute to cancer cell infiltration and invasiveness [4]. The activity of this gelatinase was evaluated by zymography in the supernatants of THP-1 cells (THP-1-CM) and M2-like macrophages previously treated with Nem, Cp5 and Cp17 (M2-CM—used as control, Nem-CM, Cp5-CM, CP17-CM, respectively). As shown in Figure 2E, MMP-9 activity increased significantly in M2-like macrophages with respect to THP-1 cells, whereas the treatment with Nem, Cp5 or Cp17 significantly reduced MMP-9 activity. In particular, Nem treatment induced a more marked reduction of MMP-9 activity, which was restored by Nem to the values observed in THP-1 cells, with respect to the two propolis samples. Taken together, these results indicate that M2-like macrophages are susceptible to Nem, Cp5 or Cp17 treatment, being the mRNA levels of some cytokines involved in M2-macrophage function and MMP-9 activity regulated by the treatment with these agents. Furthermore, it is interesting to notice that the tested concentrations (IC_50_ values/72 h, Table 2) did not cause a significant cell cycle arrest (Figure 2F), indicating that, under these experimental conditions, (i.e., low concentrations of Nem, Cp5 and Cp17 for short incubation times), the markers of M2 macrophages were influenced, but cell viability was not.

### 3.3. Nemorosone and Cuban Propolis Induce M2-Macrophages Depolarization

It is well known that macrophages are plastic cells, displaying different phenotypes and functions depending on the cellular microenvironment [28]. In the light of this observation, to determine whether Cp and Nem can influence the repolarization from M2 to M1 macrophages—or the depolarization from M2 to MΦ macrophages—we measured peculiar markers of each phenotype by qRT-PCR in different experimental conditions. Indeed, it is well-known that M2-like macrophages are characterized by a high expression of the membrane receptor CD206 and a low expression of some cytokines, as e.g., IL6 and TNFα, whereas the M1 phenotype expresses high levels of IL6, TNFα and the membrane receptor CD86 [27]. We demonstrated that a 24-h treatment with Nem, Cp5 or Cp17 (IC_50_-72 h) drastically reduced the gene expression of the M2-marker CD206. Furthermore, all treatments significantly increased the mRNA levels of the cytokines TNFα and IL6 when compared with the untreated M2-like macrophages (Figure 3).

### 3.4. Effect of Treatments Combined with M2-Conditioned Medium

**Figure 4:**
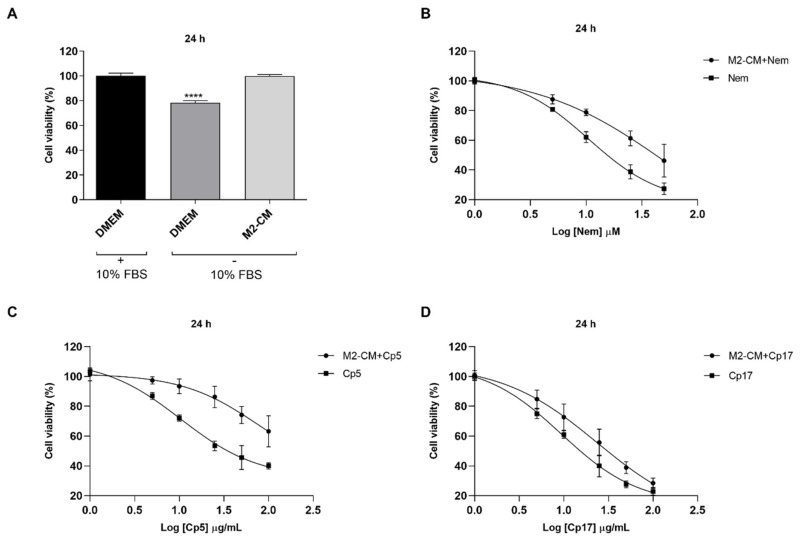
Effect of M2-conditioned medium (M2-CM) on HT-29 cell viability for 24 h. (**A**) HT-29 cells were exposed to DMEM (10% FBS), DMEM (FBS free) and M2-CM to evaluate cell viability; (**B**–**D**) HT-29 cells were exposed to M2-CM with/without increasing concentrations of Nem (**B**), Cp5 (**C**), Cp17 (**D**). Data reported as the mean ± SD of three independent experiments. **** *p* < 0.0001 vs. DMEM (10% FBS).

To investigate the effect of the conditioned medium obtained from M2-like macrophages in combination with Nem, Cp5 or Cp17 on HT-29 cell viability, the MTT assay was performed (Figure S1). We first evaluated the effect of M2-CM on HT-29 cell viability without detecting any significant difference between the M2-CM-treated cells and those cultured in DMEM with FBS supplementation. However, HT-29 cells cultured in DMEM without FBS showed a significant drop of their viability (Figure 4A). This observation indicates that M2-CM stimulated HT-29 cell viability. However, as expected, cells exposed for 24 h to M2-CM containing increasing concentrations of Nem, Cp5 or Cp17 showed a reduction of their growth in a concentration-dependent manner (Figure 4B–D), which was lower than that observed after the treatment with Nem, Cp5 or Cp17 in DMEM (Table 3). This indicates that the soluble factors present in M2-CM reduced Nem, Cp5 or Cp17-induced cytotoxicity.

**Figure 5:**
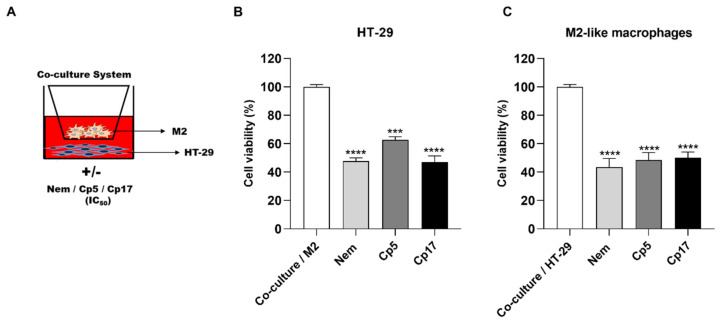
Effect of Nem, Cp5 or Cp17 (concentrations used, 24 µM, 50 µg/mL, 25 µg/mL, respectively) in co-cultures (**A**) on the viability of HT-29 (**B**) and M2-like macrophages (**C**). *** *p* < 0.001, **** *p* < 0.0001 vs. untreated cells.

These findings were confirmed by the results obtained when HT-29 cells were co-cultured with M2-like macrophages and treated with Nem, Cp5 and Cp17, respectively (IC_50_ in DMEM, Table 3) for 24 h (Figure 5A). In accordance with the findings shown in Figure 4, we demonstrated that Cp and Nem reduced the proliferation of HT-29 cells (Figure 5B) also when they were co-cultured with M2-like macrophages. A very similar effect on cell viability was observed for M2-like macrophages co-cultured with HT-29 cells because their viability was also significantly reduced (~50%) after treatment with Nem, Cp5 and Cp17 (Figure 5C).

### 3.5. Nemorosone and Cuban Propolis Inhibit Cell Migration and Invasiveness of HT-29 Cells Co-Cultured with M2-Like Macrophages

**Figure 6:**
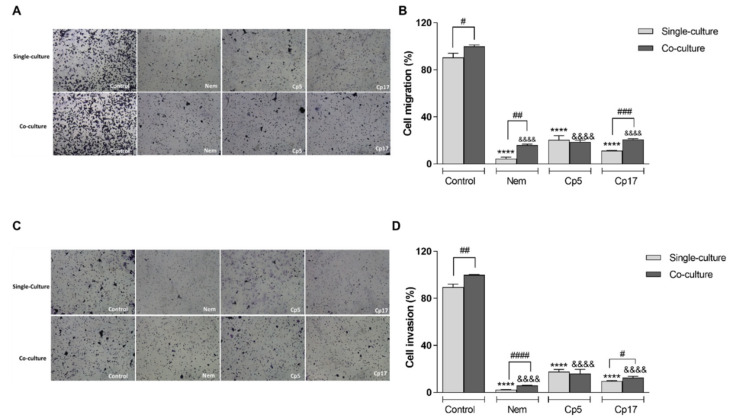
Effect of Nem, Cp5 or Cp17 (concentrations used, 12 µM, 25 µg/mL, 13 µg/mL, respectively) on (**A**,**B**) migration and (**C**,**D**) invasiveness of HT-29 cells in single culture or co-cultured with M2-like macrophages. Data are expressed as mean ± SD of three different experiments. HT-29 cells: **** *p* < 0.0001 vs. control (single culture); ^&&&&^ *p* < 0.0001 vs. control (co-culture), ^#^ *p* < 0.05, ^##^ *p* < 0.01, ^###^ p < 0.001, ^####^ *p* < 0.0001, differences between single and co-culture.

To determine the effect of Nem, Cp5 and Cp17 on migration and invasiveness of HT-29 cells, we used a Transwell system. After co-culture with differentiated THP1, HT-29 cells were assayed for their capacity of migration and invasion. The cells were treated for 24 h with Nem, Cp5 or Cp17 (half IC_50_/24 h, Table 3). As shown in Figure 6A–D, co-culturing HT-29 with M2-like macrophages led to an increase of their migration and invasiveness compared with standard conditions, indicating that M2-like macrophages play a role in the migratory and invasive behavior of HT-29 cells. Interestingly, Nem, Cp5 and Cp17-treatments decreased migration and invasion of HT-29 cells (Figure 6B,D), both in presence and absence of M2 macrophages.

### 3.6. Treatment with Nemorosone and Cuban Propolis Inhibited the Expression of Epithelial-Mesenchymal Transition Markers in HT-29 Cells Co-Cultured with M2-Like Macrophages

**Figure 7:**
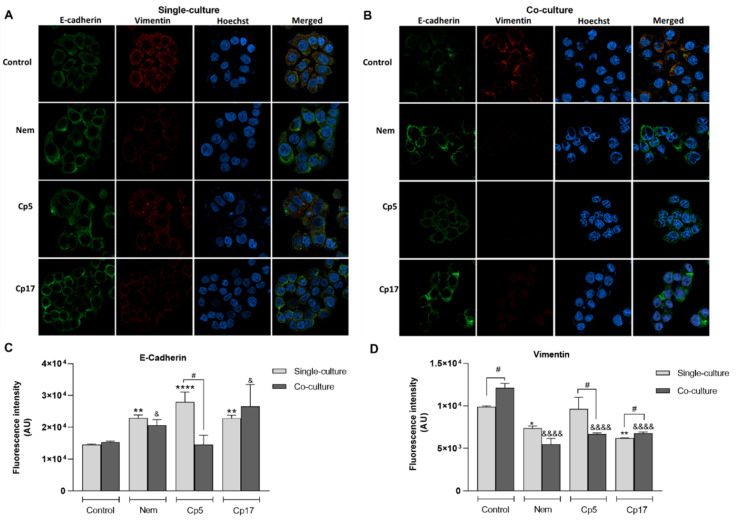
Effect of Nem, Cp5 or Cp17 (concentrations used, 12 µM, 25 µg/mL, 13 µg/mL, respectively) (half IC_50_/24 h, Table 3) on the expression of EMT-related markers E-cadherin and vimentin in HT-29 cells. (**A**) Single culture; (**B**) co-culture with M2-like macrophages. Images magnification: 40×, E-cadherin is stained in green and vimentin in red. (**C**–**D**) Nuclei are stained blue with Hoechst. Data reported as the mean ± SD of three independent experiments. * *p*< 0.05, ** *p* < 0.01, **** *p* < 0.0001 vs. control (single culture). ^&^ *p*< 0.05, ^&&&&^ *p* < 0.0001 vs. control (co-culture), ^#^ *p* < 0.05 difference between single and co-culture.

It is well known that the epithelial–mesenchymal transition (EMT) is a process underlying the tumoral progression [29], which can be modulated by M2-like macrophages within the tumor microenvironment [30,31]. To ascertain whether Nem, Cp5 and Cp17 (half IC_50_/24 h, Table 3) have an effect on EMT-associated markers, we assessed the expression of the epithelial marker E-cadherin and the mesenchymal marker vimentin in HT-29 cells co-cultured with M2-like macrophages and treated with Nem, Cp5 and Cp17. As shown in Figure 7A–D, E-cadherin expression was not influenced by the presence of M2-like macrophages in co-culture, whereas they caused a significant increase of the expression of vimentin in HT-29 cells, indicating that this macrophage phenotype could promote the mesenchymal phenotype of CRC cells. Interestingly, the expression of E-cadherin in HT-29 cells in single-culture was significantly increased by all treatments, while in HT-29 cells co-cultured with M2-like macrophage, only Nem and Cp17 induced an increase in the expression of this EMT-marker (Figure 7B,C). Hence, the expression of vimentin was significantly downregulated by Nem and Cp17 in HT-29 both co-cultured or not co-cultured with M2-like macrophages, but not by Cp5-treatment (Figure 7A,B,D).

## 4. Discussion

Considering the role of M2 macrophages in the progression of CRC, an increasing interest has been exerted by the scientific community in regulating their influence on the tumorigenicity of colon cancer cells [1]. In the present in vitro study, we investigated the effects of Cuban brown propolis and its main component Nem on a widely used colon cancer cell line (HT-29 cells [32,33]). Besides Nem, which is the major constituent of the floral resin from *Clusia rosea* Jacq. and brown Cuban propolis, we analyzed two different samples of Cp, namely Cp5 and Cp17, because Nem content and overall chemical composition of Cp are dependent on the geographical site of collection. Therefore, we aimed at ascertaining whether general conclusions could be drawn on Cp properties just considering Nem concentration in Cp samples or studies analyzing its activity, dedicated to each particular sample, are required. We here evaluated the effect of Nem and Cp on HT-29 viability and also analyzed the malignant characteristics of these cells, focusing on their crosstalk with TAMs, which are, among the various tumor-infiltrating immune cells in solid tumors, the principal population which actively participate to tumor progression [34]. Generally, TAMs exert a function similar to M2 macrophages, because the TME itself educates the infiltrating TAMs to polarize to the M2 phenotype, thereby actively participating to the suppression of antitumor immune responses and the promotion of angiogenesis [35]. Therefore, targeting TAMs infiltration and M2 polarization could be exploited as a therapeutic option for solid tumors, such as colon cancer.

As far as the M2 polarization is concerned, our findings confirmed that the combination of PMA and IL-4/IL-13 used in our study is able to induce the polarization of THP-1 monocytes into M2-like macrophages in vitro, on the basis of the expression of specific phenotypic markers. Furthermore, we demonstrated the effect of Nem and Cp samples on IL-8 and Il-10, CCL2 and VEGF. It has been reported that the M2 phenotype is characterized by a high expression of cytokines and chemokines (e.g., IL-8, IL-10, CCL2) and the growth factor VEGF [27,36]. In our study, we demonstrated that Nem, Cp5 and Cp17 remarkably inhibited the viability of M2-like macrophage in a dose-dependent manner and suppress the genetic expression of a panel of cytokines and chemokines, including *IL-8*, *IL-10*, *CCL2* and the growth factor *VEGF* peculiarly expressed by M2 macrophages. IL-8, an inflammatory cytokine, is secreted by the M2-like macrophages into the TME, and acts as a cancer progression and angiogenesis promoter [37]. Conversely, IL-10 acts as an immunosuppressant, decreasing the antitumor response of T cells, thereby facilitating tumor proliferation [38]. The chemokine CCL2 produced by TAM promotes macrophages accumulation and influences cell growth, invasion and metastasis [39,40]. Furthermore, it is well-known that the angiogenetic factor VEGF is strongly involved in the generation of cancer metastasis [41,42]. However, we measured only the mRNA expression of these cytokines after incubation with Cp and Nem, we can reasonably hypothesize that these treatments could affect the properties of M2-like macrophages and thereby alter the underlying signaling pathways playing a role in cancer progression.

To obtain further information about the targets of Nem, we also demonstrated that Nem, Cp5 and Cp17 decreased the M2-macrophage-induced MMP-9 release. M2 macrophages produce high levels of MMP-9, which is an important player in cancer progression, because it degrades the extracellular matrix [43]. Accordingly, our results showed that the mRNA expression of the cytokines *TNF-a* and *IL-6* were upregulated by Nem, Cp5 and Cp17, while the M2-like macrophage marker *CD206* was significantly downregulated, indicating that Nem, Cp5 and Cp17 exert a proinflammatory activity, which is typically associated with M1 macrophages, cells playing a proinflammation role, thereby stimulating tumor immunity and suppressing tumor progression in the TME [44]. Previous studies have shown that propolis and its components display an immunomodulatory activity and can act, depending on their concentration, on diverse pathways involved in macrophage activity and plasticity [45,46,47]. In this study, we provided the first evidence that Cuban brown propolis and Nem could reprogram M2-like macrophages. However, we are aware that this observation needs to be studied in deep and further studies are definitely required to confirm and describe in detail the mechanism(s) by which Nem regulates the dynamic transition from M2 to MΦ/M1 macrophages.

Numerous studies have demonstrated that the communication between colon cancer cells and M2-polarized macrophages generates a microenvironment suitable for tumor growth [1,48,49]. Accordingly, we observed that M2-conditioned medium induced HT-29 cell proliferation, indicating that M2-CM contains soluble factors able to induce cell proliferation. Consistent with these results, we demonstrated that HT-29 cells exhibited an increase in cell viability when co-cultured with M2-macrophages and this increase was counteracted by the treatment with Nem, Cp5 and Cp17. Clinical and experimental studies have suggested that natural products can influence macrophage polarization, in particular by inhibiting the expression of M2 peculiar genes promoting tumor proliferation, migration, invasion and angiogenesis [5,50,51]. Therefore, on the basis of our results, demonstrating the modulation of M2-associated genes and M2-depolarization induced by Cp and Nem, we hypothesize that they exhibit the observed inhibitory effect on HT-29 cell growth by suppressing the production of soluble factors involved in the communications between TAMs and cancer cells.

The activation of epithelial mesenchymal transition (EMT) is a complex process that involves different phenotypic changes, resulting in increased migration, invasiveness and metastatic properties of tumor cells [29,52]. In particular, during the EMT, invasive cancer cells are triggered to replace epithelial-related genes with mesenchymal-related ones, leading to the loss of cell–cell adhesions, apical-basal polarity and epithelial markers as E-cadherin. Furthermore, it has been demonstrated that M2 macrophages promote the EMT of cancer cells [53,54]. Our results are in line with these previous observations since we observed an increase of HT-29 migration and invasion when these cells were co-cultured with M2 macrophages. Notably, we also demonstrated that the increase of migratory and invasive capacity of HT-29 cells induced by M2-macrophages was counteracted by treatments with Nem, Cp5 and Cp17. Accordingly, we observed that Nem, Cp5 and Cp17 altered the expression and function of the EMT-markers E-cadherin and vimentin. In particular, we observed that HT-29 cells co-cultured with M2-macrophages exhibited an increase in E-cadherin and a decrease in vimentin expression following Nem, Cp5 and Cp17 treatment. It has to be noticed that, in our experimental conditions, the expression of E-cadherin was unchanged when HT-29 cells were co-cultured with M2-macrophages, whereas the expression of vimentin was significantly increased. It has already been demonstrated that TAMs play an important role in EMT by decreasing E-cadherin and increasing vimentin, thereby contributing to the invasiveness and metastatic properties of tumor cells [52,55]. However, the loss of E-cadherin function, which leads to the destabilization of adherent junctions, is controlled by various molecular mechanisms, including silencing mutations in the E-cadherin gene or its transcriptional repression [56]. Therefore, the experimental conditions of our study could be responsible for the absence of the M2-macrophage effect on E-cadherin expression. After the demonstration of the antimetastatic potential of CP and Nem [17], the present findings indicate that Nem and two different propolis samples (Cp5 and Cp17) could also inhibit, with slight differences, the M2-induced mesenchymal morphologic changes of HT-29 cells.

## 5. Conclusions

In conclusion, our findings show for the first time that Cuban brown propolis and its main component nemorosone influence the crosstalk between colon cancer cells and TAMs, by inhibiting the viability of M2-like macrophages and modulating their polarization. All these events concur in impairing the proliferation, migration and invasiveness of HT-29 cells. Accordingly, Cp and Nem downregulate the expression of EMT-related markers. Our results, which are consistent with previous observations and confirm that Nem is the bioactive compound responsible for Cp efficacy, shed light on a promising application of propolis. However, further studies addressing with detailed investigations the molecular mechanisms, as well as the in vivo efficacy of Nem contained in Cp, will help to provide a complete picture of Cp and Nem effects.

## Figures and Tables

**Table 1:** Sample nemorosone (Nem) concentrations.

Propolis Concentration (µg/mL)	Nemorosone Concentration (µM)	
	Cp5	Cp17
6.25	2.7	4.2
12.5	5.4	8.4
25	10.8	16.9
50	21.6	33.7
100	43.2	67.4

**Table 2:** IC_50_ values of Nem and Cp samples.

Time	Nem (µM)	Cp5 (µg/mL)	Cp17 (µg/mL)
72 h	21.35 ± 8.34	44.34 ± 2.8	16.04 ± 2.96

Data expressed as mean ± S.D.

**Table 3:** IC_50_ values of Nem and Cp samples. Treatment was performed in DMEM or in combination with M2-CM.

Sample	DMEM (FBS Free)	M2-CM
Nem (IC_50_)	23.55 ± 4.9 (µM)	41.92 ± 7.3 * (µM)
Cp5 (IC_50_)	49.71 ± 2.3 (µg/mL)	N.C.
Cp17 (IC_50_)	25.51 ± 3.3 (µg/mL)	35.35 ± 3.9 ^£^ (µg/mL)

Data expressed as mean ± S.D. * *p* < 0.05 vs. Nem (DMEM–FBS free), ^£^ *p* <0.05 vs. Cp17 (DMEM–FBS free). N.C., non-calculable.

## Supplementary Materials

The following are available online at https://www.mdpi.com/2072-6643/12/7/2040/s1. Figure S1. Schemes of treatments used to evaluate MMP9 activity in different conditioned media (A) and HT29 cell viability after 24 h treatment with M2 medium with or without Nem, Cp5 and Cp17 (B). Table S1. Primers used in this study for qPCR experiments.
