# Supplementary material for: Cuban Brown Propolis Interferes in the Crosstalk between Colorectal Cancer Cells and M2 Macrophages
Source: Nutrients. 2020 Jul 9;12(7):2040. doi: 10.3390/nu12072040 (PMC7400951; doi:10.3390/nu12072040)
Supplement: Supplementary file 1 [file nutrients-12-02040-s001.pdf]

Figure S1. Schemes of the treatments used to evaluate MMP9 activity in different conditioned media (A) and HT29 cell viability after 24 h treatment with M2 medium with or without Nem, Cp5 and Cp17 (B).

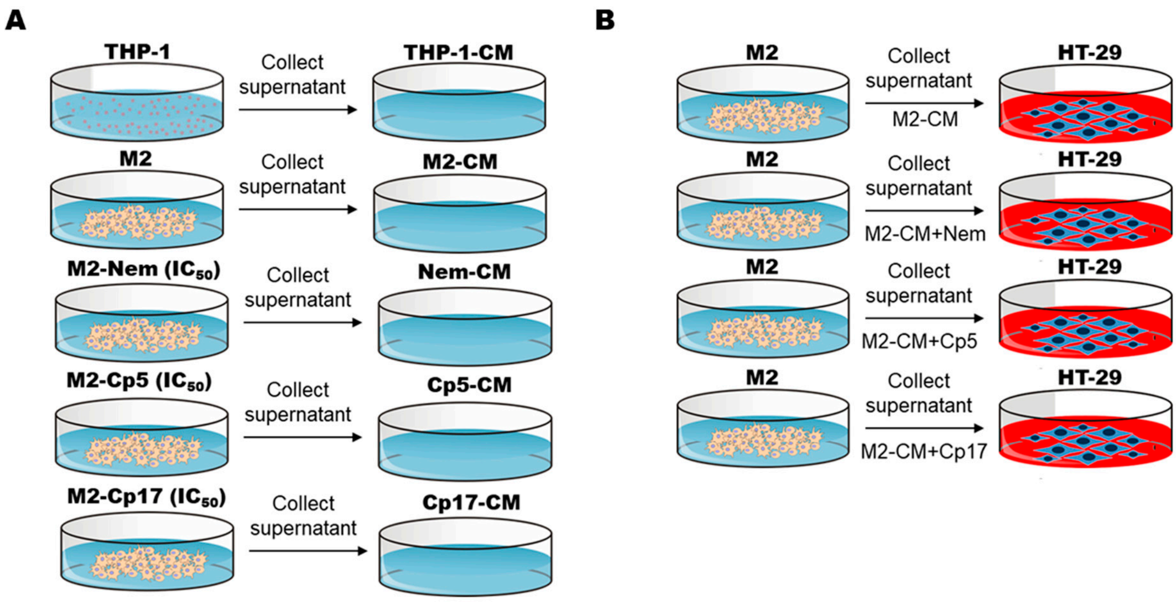

Table S1. qRT-PCR primers used in this study.

Gene	Sequence forward (F)	Sequence reverse (R)
IL6	ACTCCTTCTCCACAAGCGCC	TGTGGGGCGGCTACATCTT
IL8	AAACCACCGGAAGGAACCAT	CCTTCACACAGAGCTGCAGAAA
TNFα	AAGCCTGTAGCCCATGTTGT	GAGGTACAGGCCCTCTGATG
CCL5	CCTCATTGCTAGGCCCTCT	GGTGTGGTGTCCCGAGGAAT
IL10	AAGACTTTCTTTCAAATGAA	GTTTTCACAGGGAAGAAATC
CCL2	GCTCATAGCAGCCACCTTCA	GGACACTTGCTGCTGGTGAT
VEGF	ATGGCAGAAGGAGGAGGGCA	ATCGCATCAGGGGCACACAG
CD206	ACCTTCACAAGTATCCACACCATC	CTTCATCACCACACAATCCT
GAPDH	ACATCAAGAAGGTGGTGAAGCA	GTCAAAGGTGGAGGAGTGGGT
